# Does a Pre-Training Program Influence Colonoscopy Proficiency during Fellowship?

**DOI:** 10.1371/journal.pone.0164360

**Published:** 2016-10-20

**Authors:** Duk Hwan Kim, Soo Jung Park, Jae Hee Cheon, Tae Il Kim, Won Ho Kim, Sung Pil Hong

**Affiliations:** 1 Digestive Disease Center, CHA Bundang Medical Center, CHA University School of Medicine, Seongnam, Korea; 2 Department of Internal Medicine and Institute of Gastroenterology, Yonsei University College of Medicine, Seoul, Korea; University Hospital Llandough, UNITED KINGDOM

## Abstract

**Objectives:**

The objective of this study was to determine whether a pre-training program influences the entire learning process and overall proficiency of colonoscopy during fellowship.

**Methods:**

From March 2011 to February 2013, a total of 28 first-year gastrointestinal fellows were trained in colonoscopy at a single tertiary center. Before entering their fellowship training, all fellows were board certified in internal medicine, but had no experience performing a full colonoscopy. Endoscopic quality indices were prospectively measured throughout the first training year and were compared between two groups, “pre-trained” fellows (n = 14), who had more than 100 cases of upper endoscopy experience and colonoscopy observation before starting their fellowship, and the “not pre-trained” group (n = 14), who had less experience.

**Results:**

A total of 15,494 colonoscopies were evaluated and 5,411 were screening colonoscopies. There were no significant differences in the overall quality index between the pre-trained and not pre-trained groups. However, the improvement in the adenoma detection rate (ADR) from the first half of the year to the latter half was significantly higher for the pre-trained group compared to the not pre-trained group (28.6% to 34.5% vs. 36.7% to 28.3%, respectively, *P* = 0.007). Multivariate analysis showed that pre-training before learning colonoscopy was the only significant factor for high ADR in the second half of the year (11.666 ± 4.251 [B±SE], *P* = 0.012).

**Conclusion:**

Sufficient observation of colonoscopy and experience of upper endoscopy before colonoscopy training might facilitate improvement of fellows’ manual and cognitive colonoscopic skills during the learning period.

## Introduction

Colonoscopy serves an important role in diagnosing and preventing colorectal cancer (CRC). It has been established that screening with colonoscopy or flexible sigmoidoscopy significantly reduces the incidence and mortality of CRC [1–5]. Because preventing this cancer is based on the removal of adenomatous polyps during screening colonoscopy, the importance of comprehensive quality improvement regarding colonoscopy has been emphasized [6]. There have been abundant investigations on quality of colonoscopy such as precise indications for colonoscopy, appropriate bowel preparation and endoscopic skill competence [7–11]. However, when analyzing the ability of the operator, withdrawal time and adenoma detection rate (ADR) are generally accepted as colonoscopy quality indicators [12–14].

Because colonoscopy involves integrated performance that requires both technical and cognitive abilities, the outcome of the procedure is strongly influenced by the training and experience of the operator [15–18]. Several guidelines [19–23] have proposed the minimal number of endoscopic procedures required to obtain appropriate competence and some studies [24,25] have sought to develop programs to improve the learning curve of endoscopic examination. However, no simple intervention has been identified as an effective single measure that can be easily implemented to improve competence. Furthermore, current investigations have focused on the technical aspects of colonoscopy, such as cecal intubation rates or withdrawal time [23,26,27]. Considering cognitive competency, endoscopic trainers might lead fellows to concern their improvement of quality indicator such as ADR and withdrawal time.

Each trainee has an individual learning pace. Therefore, it is necessary to identify the factors associated with improvement of a trainee’s capability during training. However, how prior knowledge and experience of trainees affect technical and cognitive progress during colonoscopy training remains unclear. The aim of the present study was to determine whether a pre-training program influences the entire colonoscopy learning process of and helps trainees achieve adequate proficiency including ADR.

## Materials and Methods

### Subjects

From March 2011 and February 2013, a total of 28 first-year GI fellows (14 fellows in each year) were enrolled in the prospective observational study at Severance Hospital, Seoul, Korea. The endoscopy unit of Severance Hospital was certified for endoscopy training by the Korean Society of Gastrointestinal Endoscopy. Before starting colonoscopy training, all fellows were board certified in internal medicine. To compare colonoscopy outcomes, fellows were subdivided into two groups according to their participation in pre-training program. The pre-training program of endoscopy was provided to subspecialty residency trainee of gastroenterology in the hospital as a part of the residency training. The minimal criteria of completing pre-training program in this study was defined as a course of more than 100 cases of esophagogastroduodenoscopy and more than 200 cases of colonoscopy observation over a period of 12 months. To classify fellows from other hospitals, individual interviews were done before training and fellows who met those criteria were regarded as pre-training group. In total, there were 14 fellows included in the pre-trained group and 14 fellows were in the not pre-trained group. Written Informed consent regarding procedure and comprehensive data collection was obtained at patient’s visit to the gastroenterology outpatient clinic before colonoscopy. Informed consent for data analysis was not obtained as the study was designed as an observational study with minimal risk of patient’s safety, which was approved by the Institutional Review Board of Severance Hospital. (protocol number 4-2013-0915)

### Colonoscopic examination

All colonoscopies were performed under the guidance of attending staff. Basically, fellows were allowed to attempt full colonoscopy if possible. Oral advice from the staff was given when the fellow had difficulties in reaching the cecum. However, staff were able to take over the procedure in complicated cases at their own discretion. Intramuscular injection of pethidine hydrochloride (25 mg) was given as a pre-procedural treatment if the condition of the patient was allowed. Endoscopist-directed sedation was provided using intravenous injection of propofol (1 mg/kg) or midazolam (3–5 mg) before starting the examination. If needed, intra-procedural bolus injections of propofol (20 mg) were added by an assistant. Blood pressure, electrocardiography and oxygen saturation of the patient were monitored during the procedure. Bowel preparation was performed using single or split dose of polyethylene glycol (4 liters). Failed cases caused by poor preparation or disease, such as cancer or severe colitis, were not included in this study. All colonoscopies were performed using CF-Q260AI, CF-H260AI or PCF-Q260AI colonoscopes (Olympus Optical Co, Ltd, Tokyo, Japan).

### Quality indicators

To evaluate the colonoscopic skills of the fellows, the outcomes from the first half of the year were compared with those of the last half of the year. ADR and withdrawal time were regarded as cognitive quality indicators, while success rate and insertion time were used as technical quality indicators. ADR was defined as the proportion of patients with one or more pathologically confirmed adenoma during screening colonoscopy in patients aged 50 and above. Withdrawal time was calculated as the elapsed time between cecal intubation and the completion of colonoscopy in cases without lesions such as polyps or colitis. Similarly, insertion time was calculated by the time interval between the initiation of the examination and reaching the cecum from cases in which biopsy or polypectomy were not undertaken during insertion. The success rate was defined as the proportion of cases in which a close picture of the appendix orifice was obtained compared to all colonoscopies. Because subjective measurement of staff’s intervention was not possible, insertion time and success rate represented cooperative outcomes of fellows and staff doctors. Each quality indicator at time period (a whole year, first half of the year, and last half of the year) was calculated separately. The mean value of each quality indicator was regarded as a kind of individual score for statistical analysis. The quality of bowel preparation was categorized into ‘excellent’, ‘good’, ‘fair’ or ‘poor’. Adequate bowel preparation was defined as the proportion of cases in which colon cleansing was regarded as more than ‘good’ from all cases.

### Statistical analysis

Continuous variables were compared using the Student’s t-test and presented as the mean (±standard deviation) in this paper. Dichotomous or nominal categorical variables are compared with the use of the chi-square test with normal approximation or Fisher’s exact test, as appropriate. To compare the improvement of quality indicators during training period between two groups, the variance between the first half of the year and the last half of the year of several variables including quality indicators were analyzed by two-way repeated measures analysis of variance (ANOVA). The interaction effect of time (first and last half of the year) and group (pre- or not pre-trained) from within-subject effects was considered as those difference of trend between groups. The ADR of the last half of the year was considered as the final indication of competency of the fellows. To identify factors associated with a high ADR in the last half of the year, multivariate analysis was performed using a multiple linear regression method. *P*-values less than 0.05 were considered statistically significant. All statistical analyses were performed using PASW statistics version 18.0 for Windows (IBM SPSS IBM Corporation, NY, USA)

## Results

### One-year outcomes of colonoscopy

A total of 26,987 colonoscopies were performed in our institution. Among those cases, 15,494 (57.4%) were performed by fellows and 5,411 (34.9%) were for the purpose of colorectal cancer screening. From the one-year outcomes of all fellows, mean insertion time was 821.3 ± 192.2 seconds and the overall success rate was 96.6%. Mean withdrawal time was 501.0 ± 70.3 seconds. Among screening colonoscopies the ADR was 31.5%. Bowel preparation was adequate 83.8% of cases.

Among 15,494 cases, the pre-trained group (14 fellows) performed 7,330 colonoscopies (47.3%), and the not pre-trained group (14 fellows) performed 8164 colonoscopies (52.7%; Table 1). For screening colonoscopy cases, age, sex, and bowel preparation were not different between the two groups. Insertion time tended to be shorter in the pre-trained group than the not pre-trained group, but this was not significant (751.6 sec vs. 891.0; P = 0.053). Other quality indicators were not different between the two groups (Table 1).

**Table 1:** Overall colonoscopic outcomes of fellowship trainees.

	Pre-trained group (14 fellows)	Not pre-trained group (14 fellows)	*P*-value
Total cases	7330	8164	0.346
Screening cases	2870	2541	0.403
Mean age of patients	57.0 ±1.7	57.5 ±1.9	0.491
Proportion of male sex (%)	49.0	49.6	0.814
Adequate bowel preparation (%)	83.5	84.1	0.831
Insertion time (sec)	751.6 ±131.8	891.0 ±221.1	0.053
Withdrawal time (sec)	494.0 ±69.5	508.1 ±73.0	0.605
Success rate (%)	96.9	96.3	0.401
ADR (%)	31.3	31.8	0.861

ADR, adenoma detection rate

### Competency improvement of the fellows

**Fig 1:**
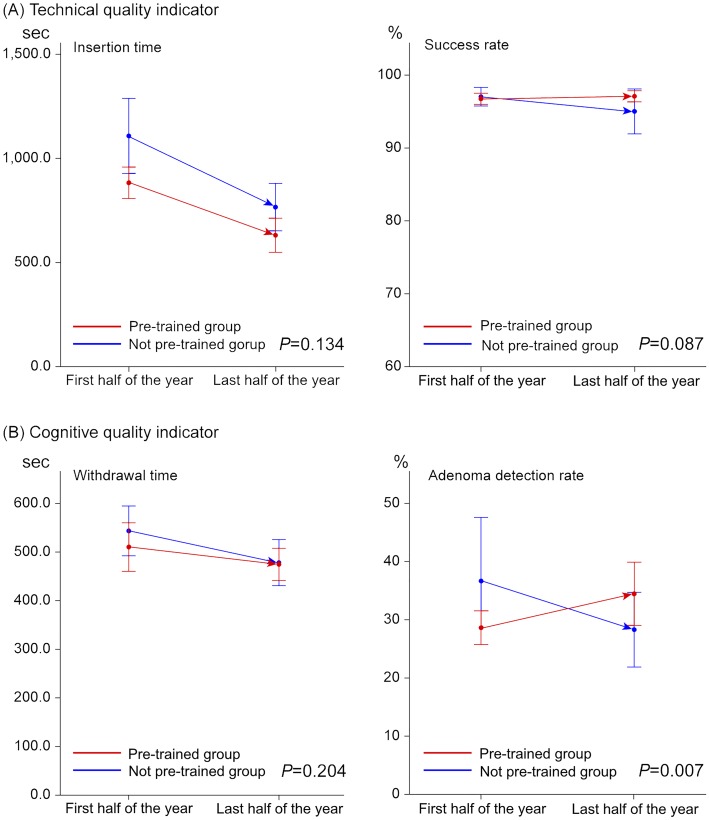
Comparison of outcome variation over time between pre-trained and not pre-trained groups.

In the first half of the year, mean insertion time was significantly shorter in the pre-trained group than the not pre-trained group (882.7 ± 131.1 seconds vs. 1,107.9 ± 312.2 seconds, *P* = 0.023; Table 2). Mean insertion time decreased to 630.9 ± 141.7 sec in the pre-trained group and 766.1 ± 196.7 sec in the not pre-trained group during the last half of the year, which was a finding with statistical significance (*P* = 0.047). However, the improvement between the first half and the last half of the year in each group was not statistically significant (-251.8 seconds in the pre-trained group and -341.8 seconds in the not pre-trained group, *P* = 0.134). Success rates of the pre-trained group and the not pre-trained group were 96.7 ± 1.3% and 97.0 ± 2.2% (*P* = 0.673) in the first half of the year and 97.1 ± 1.3% and 95.0 ± 5.3% (*P* = 0.169) in the last half of the year, respectively. And their variances by the lapse of time between groups did not show statistical significance (*P* = 0.087). The withdrawal time in the first and last half of the year was 510.3 ± 86.3 sec and 474.6 ± 57.7 sec in the pre-trained group, and 543.6 ± 88.8 sec and 478.3 ± 82.2 sec in the not pre-trained group, respectively. And their variances along the training period were not significantly different between the two groups (*P* = 0.204). In the first half of the year, the ADR was 28.6% in the pre-trained group and 36.7% in the not pre-trained group (*P* = 0.136); however, the ADR of the pre-trained group increased to 34.5% in the last half of the year, while the ADR in the not pre-trained group dropped to 28.3%. Change in the ADR as training time progressed was significantly different between the two groups (*P* = 0.007; Table 2 and Fig 1)

**Table 2:** Comparison of colonoscopic quality indicators of fellowship trainees between the first half of the year and last half of the year.

	Pre-trained group (14 fellows)	Not pre-trained group (14 fellows)	*P*-value
First half of the year			
Total cases	327.8 ±137.4	292.2 ±156.7	0.529
Insertion time (sec)	882.7 ±131.1	1107.9 ±312.2	0.023
Withdrawal time (sec)	510.3 ±86.3	543.6 ±88.8	0.323
Success rate (%)	96.7 ±1.3	97.0 ±2.2	0.673
ADR (%)	28.6 ±5.0	36.7 ±10.6	0.136
Last half of the year			
Total cases	193.9 ±111.2	290.9 ±120.5	0.036
Insertion time (sec)	630.9 ±141.7	766.1 ±196.7	0.047
Withdrawal time (sec)	474.6 ±57.7	478.3 ±82.2	0.892
Success rate (%)	97.1 ±1.3	95.0 ±5.3	0.169
ADR (%)	34.5 ±9.4	28.3 ±11.1	0.125

ADR, adenoma detection rate

### Factors affecting high ADR in the last half of the year

Multivariate analysis was performed to reveal factors associated with high ADR in the last half of the year of fellowship. Insertion time, withdrawal time and success rates in the last half of the year were considered as indicators of the final ability of the fellow. The proportion of adequate bowel preparation in the last half of the year was also included in analysis. Pre-training before fellowship was the only independent factor associated with high ADR in the last half of the year (B±SE = 11.666±4.251 and *P* = 0.012). (Table 3)

**Table 3:** Factors associated with higher adenoma detection rate in the last half of the year^a^.

	B±SE^b^	B^c^	*P*-value
Pre-training	11.666±4.251	0.561	0.012
Longer withdrawal of last half of the year	0.057±0.028	0.372	0.059
Shorter insertion time of last half of the year	0.014±0.011	0.235	0.218
Higher success rate of last half of the year	-1.658±0.898	-0.620	0.078
Adequate bowel preparation of last half of the year (more than good)	0.009±0.277	0.010	0.973

^a^ R^2^ = 0.399, F = 2.919, *P* = 0.036

^b^ B and SE denote the variable estimate and standard error, respectively.

^c^ β denotes the standardized estimate.

## Discussion/Conclusions

Several colonoscopy quality issues have been discussed since the introduction of endoscopic screening for CRC. Although the comprehensive quality of colonoscopy can be influenced by multiple factors, thus far the competency of an endoscopist remains the most important component of endoscopic examination.

The effort to improve individual colonoscopic proficiency has to start in colonoscopy training. However, existing investigations of colonoscopic training were limited to the technical aspects of endoscopy, such as success rate and withdrawal time [23,28–31]. As advancements in insertion techniques and tools, such as variable stiffness scopes, are developed, cecal intubation itself is no longer an issue. In our study, the lowest individual success rate of first half of the year was 92.4%. Despite longer insertion times, beginners were able to reach the cecum under the guidance of a senior physician after a short initial training period. Therefore, when considering both technical and cognitive aspects, achieving adequate ADR and short insertion time should be key targets of colonoscopy training.

Traditionally, a minimal number of endoscopies was used as an adequate indicator of colonoscopy training [19,29,32]. However, the number of procedures cannot represent overall competency because colonoscopic outcome is affected by complex factors including skill and knowledge of the individual endoscopist. A recent study that investigated cecal intubation rates of 297 trainees revealed that the distribution of colonoscopic completion rates varied among fellows [32] and the adequate number of procedures for colonoscopic competency appeared to vary depending on the study and ranged from 100 to 500 cases [33]. Moreover, an investigation that studied potential factors associated with ADR showed that annual case volume did not correlate with ADR [17].

Some studies [24,25] reported that educational intervention enabled physicians to improve their ADRs. Prior studies focused on self-directed learning; however, each fellow had a different rate of skill improvement [22], and every training center has their own educational intervention methods. Generally, different progress rates are thought to be a matter of the talents of the individual fellows. Nevertheless, in the context of teaching and improving the quality of colonoscopy training, determining and increasing the talents of fellows are essential. Very little is known about what qualifies as sufficient training prior to learning the skills necessary to perform colonoscopies. A multi-center investigation reported that experience with upper gastrointestinal endoscopy and colonoscopic seminar attendance was associated with shorter cecal intubation times [23]. However, favorable influence of those educations was restricted on very early period of colonoscopy training. Moreover, cognitive quality indicators such as ADR were not evaluated in the study.

The current study revealed that mean insertion time was significantly shorter when performed by fellows who had more experience with upper endoscopy and observation of colonoscopic examination than those who did not. This difference was consistently shown from the beginning to the end of training. From the perspective of manual ability, it is inferred that fellows who had experience in the manipulation of upper gastrointestinal endoscopes had better endoscopic orientation and insertion techniques. Considering the invasive nature of colonoscopy, we postulate that sufficient prior experience using an endoscope allows for effective training by reducing unnecessary discomfort in patients. A recent study similarly reported that experience with flexible sigmoidoscopy was independently associated with attaining adequate cecal intubation rates [32]. However, another technical indicator, mean success rate, was not significantly different between the two groups. Additionally, cognitive indicators such as mean ADR and withdrawal time did not vary significantly.

One interesting finding of the present study was the increment of mean ADR of the pre-trained group, while the ADR of the not pre-trained group showed a trend toward a decrease in ADR throughout the year. Although differences in mean ADR between the two groups were not significantly different, variation in training progress was. One explanation is that the pre-trained group was able to concentrate on the quality of inspection as the technical burden was lessened compared to in the not pre-trained group. Similarly, a prospective multicenter study on the learning curve of colonoscopy fellows reported no significant improvement in polyp detection rates during the training period while success rates and insertion times consistently improved over time [23]. Other possibilities may include sufficient baseline ADRs and withdrawal times. The lowest mean ADR and the longest withdrawal time in the first half of the year were 28.6% and 510 seconds, respectively. It is presumed that fellows were fairly careful about their procedures in the early training period. Therefore, the learning effect on cognitive ability might be restricted in first half of the year. Despite of the absence of statistical significance, numerical high ADR and withdrawal time in the early training period of not pre-trained group were able to be explained in the same manner.

The present study revealed that pre-training was the only independent factor of high ADR in the latter half of the year-long training period. Longer withdrawal time and other technical indicators such as shorter insertion time and high success rate were not significantly associated with high ADR achievement. Fellows in this study spent an adequately long time (370.1 seconds in the fellow who had the shortest mean withdrawal time) during withdrawal, therefore, influence of inspection time on endoscopic quality might be limited.

This study has several limitations including a relatively small number of study subjects, a poorly organized pre-training program, and frequent involvement of senior endoscopists during the procedures. Although we conducted minimal criteria for classifying fellows before training, there might be potential possibilities regarding selection bias because of non-randomized study design. And another important limitation of the study is the intervention of trainers. Due to regulations of the hospital, the complete restriction of trainers’ involvement during training was not possible. Therefore, the success rate and insertion time of this study was not able to represent true outcomes of fellows. Subjective analysis of trainers’ involvement was not possible because each trainer had different inclination regarding education of endoscopy. However, we thought frequent interventions might affect colonoscopic outcomes including insertion times negatively. One trainer generally taught two fellows at a time, and usually focused on insertion period because of patient’s safety. Therefore, a fellow is largely responsible for detecting the lesion. However, the trainers sometimes followed the fellow’s procedure from beginning to end if the time permits. ADR in this study may be influenced by those interventions of trainers. Most of all, we cannot analyze quality indicators of the fellows based on an interval shorter than 6 months. A first year fellow participates in lower GI section twice a year on the average. Although two times of lower GI section was usually placed one in the first and one in the last half of the year roughly, fellows did not perform the same number of colonoscopy examinations over the course of the year due to variation in the overall GI training program of the hospital. Especially, analyzing ADR by consecutive short intervals was not possible because screening cases for ADR calculation were relatively small and unevenly distributed through the year.

However, the current study demonstrates that fellows who had sufficient experience with endoscope manipulation and observation of expert examinations showed significantly better technical outcomes. In addition, pre-trained fellows also consistently retained their cognitive skill increment through the training period. Therefore, a systematic educational program before training in colonoscopy might be needed to improve both technical and cognitive abilities. In conclusion, sufficient colonoscopy observation and upper endoscopy experience before training may enhance the technical and cognitive skills of fellows, as indicated by the high ADR related to prior experience with endoscopy.
